# A cross-sectional study exploring the relationship between symptoms of anxiety/depression and P50 sensory gating in adult patients diagnosed with chronic fatigue syndrome/myalgic encephalomyelitis

**DOI:** 10.3389/fnins.2023.1286340

**Published:** 2024-01-05

**Authors:** Xinyi Liu, Sitong Liu, Runtao Ren, Xue Wang, Chunyu Han, Zhandong Liu

**Affiliations:** ^1^Department of Neurology, Health Care Centre, Beijing Friendship Hospital, Capital Medical University, Beijing, China; ^2^Department of Neurology, Beijing Friendship Hospital, Capital Medical University, Beijing, China

**Keywords:** chronic fatigue syndrome/myalgic encephalomyelitis, anxiety, depression, auditory evoked potential P50, functional neurological disorder

## Abstract

**Introduction:**

Chronic fatigue syndrome (CFS) is a clinical disease that affects multiple body systems. It is characterized by persistent or recurring fatigue, which may be linked to immune, neuroendocrine, and energy metabolism dysfunctions. Individuals with CFS may experience pain, sleep disorders, anxiety, and depression. This research analyzed the fundamental characteristics of anxiety/depression symptoms in patients with CFS and investigated the association between these symptoms and the P50 SG (sensory gate) ratio.

**Methods:**

Two hundred and forty-nine subjects fulfilled the CDC-1994 criteria for CFS and were included in the study. The subjects successively completed the Symptom CheckList-90-Revised (SCL-90-R), Hamilton Anxiety Rating Scale-14 (HAMA-14), and Hamilton Depression Rating Scale-24 (HAMD-24). Auditory-evoked potential P50 were measured using the 128-lead-electroencephalograph.

**Result:**

According to HAMA and HAMD, 17.3% (*n* = 43) of the patients did not exhibit anxiety/depression, with a threshold score of 7 and 7 for HAMA and HAMD. When the threshold score was 14 and 20 respectively, 43.3% (*n* = 108) of the patients did not exhibit anxiety/depression. The SCL-90-R results indicated that 69.5% (*n* = 173) of these individuals with the score arranging from 0 to 160 did not present mental problems. There was a correlation between somatization scores and P50 SG ratio in the overall sample and no anxiety or depression (NAOD) group delimited by 14 and 20, respectively, (*p* < 0.05). Regression analysis showed that anxiety and depression were risk factors associated with an abnormal P50 SG ratio.

**Discussion:**

A significant correlation exists between the P50 SG ratio and clinical symptoms such as fatigue, anxiety, and depression. Abnormalities in brain function among patients with CFS may play a crucial role in the pathogenesis of the condition, leading to their classification as being prone to functional neurological disorders. The P50 SG ratio cannot be used as a diagnostic marker for CFS but show some significance on the mechanism, classification, treatment, and prognosis of CFS.

## 1 Introduction

Chronic fatigue syndrome/myalgic encephalomyelitis (CFS/ME) is a debilitating disease involving persistent or recurrent fatigue. The persistent and unmitigable symptoms of CFS have seriously affected the daily life and work of patients. Although many clinicians consider CFS a neurological disease, its complex features intersect with those of other systemic diseases, such as irritable bowel syndrome, hyperventilation syndrome, and polymyalgia rheumatica (Teodoro et al., 2018). There is considerable controversy over CFS/ME, as some scholars consider it a mental disorder similar to anxiety and depression (Manu et al., 1988; Roy-Byrne et al., 2002). This controversy stems from the fact that it is primarily diagnosed based on symptoms, and the United States CDC 1994 diagnostic criteria are most widely used (Fukuda et al., 1994). According to research reports, the global incidence rate of CFS has shown a rapid upward trend approximate to 1% in recent years (Prins et al., 2006). CFS is challenging to treat and highly disabling, severely impacting patients’ quality of life. With the increase in social pressure and the influence of the environmental, infectious diseases, and other factors, the incidence of CFS in China has significantly increased (Haider et al., 2023), with its persistent and unmitigable symptoms affecting people’s daily activities and work. Hence, CFS is a significant problem endangering social public health.

The fatigue dimension shown by patients with CFS has physical and mental implications. The main manifestations are anxiety, depression, irritability, and emotional instability. Its mechanisms may be related to immunity, neuroendocrine, and energy metabolism (Carruthers et al., 2011; Clayton, 2015). For a long time, the relationship between CFS and anxiety/depression could not be explained, as the three have differences and similarities (Sáez-Francàs et al., 2012; Wright et al., 2021). Studies have shown that CFS, anxiety, and depression may show the same trend of oxidative stress immunity, inflammation, and endocrine changes (Kennedy et al., 2005). However, the degree of biological changes in CFS, anxiety, and depression significantly differ (Shungu et al., 2012). Cockshell et al. reported that fatigue in patients with CFS was not associated with depression (Cockshell and Mathias, 2013). However, these conditions undergo dynamic changes throughout their course, necessitating the need for ongoing patient monitoring. The underlying mechanism linking fatigue with anxiety and depressive symptoms in patients with CFS warrants further investigation.

Abnormal brain function has been reported in both anxiety/depression and CFS, and significant progress in this aspect has been made with the development of neuroimaging technology (Barnden et al., 2015; Klumpp and Shankman, 2018). Some scholars believe that “cortical diencephalic syndrome,” a stress-induced abnormality in brain function, can be classified as a subtype of CFS. CFS patients exhibit distinct cerebral morphology, cerebral blood flow (CBF), cerebral functional connectivity, and cerebral metabolism on MRI. Numerous imaging studies have confirmed reduced CBF and abnormalities in gray and white matter signals among CFS patients. Currently, several scholars employ multimodal MRI technology to investigate the correlation between symptoms and changes in brain function, as well as systematically evaluate the effects of pharmacological, cognitive, physical, and other treatments. Above those have laid the groundwork for further research on whether the pathogenesis of CFS is associated with abnormal brain function (Xue and Liu, 2015).

Therefore, using objective indicators closely related to brain function to measure CFS and anxiety/depression symptoms is reasonable. Information processing by the human brain is regularly transmitted from the lower to the higher central nervous system, and the average human brain has a selection and filtering process for external stimulus information called sensory gating (SG). The P50 auditory evoked potential is a widely used neuroelectrophysiological examination method in research for the detection of SG, especially related with cognitive domain and emotional disorders (Harrison et al., 2019). However, there is still a lack of using P50 SG in the research field of CFS. Therefore, we are interested in checking abnormal brain function in CFS patients by examining the correlation between symptoms of anxiety or depression with the P50 SG ratio. Furthermore, we aim to explore the usage of this indicator for diagnosis and prognostic assessment. The findings of this study may provide more information for the potential of the P50 SG ratio as an objective, non-invasive diagnostic tool.

## 2 Materials and methods

### 2.1 Participants and inclusion/exclusion criteria

For this three-year cross-sectional study (from August 2019 to August 2022), data from patients with CFS at the Neuroscience Clinic of the Department of Neurology at Beijing Friendship Hospital was collected. The Human Research Ethics Committee of Beijing Friendship Hospital, affiliated with Capital Medical University, approved the study. Written informed consent was obtained from all eligible and enrolled patients.

**Figure 1:**
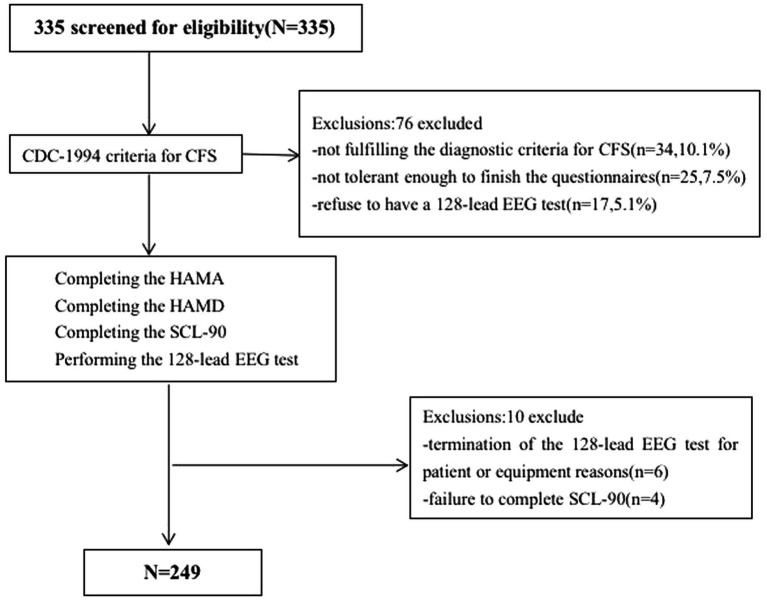
Web diagram of the study sample. CFS, chronic fatigue syndrome; HAMA, Hamilton Anxiety Rating Scale; HAMA, Hamilton Depression Rating Scale; SCL-90-R, Symptom CheckList-90-Revised Scale.

The inclusion criteria were (Teodoro et al., 2018) individuals 14–70 years old (Roy-Byrne et al., 2002) CFS diagnosed according to the CDC-1994 criteria (Manu et al., 1988) absence of fatigue due to other causes, including encephalitis, stroke, brain tumor, diabetes or metabolic syndrome (Fukuda et al., 1994) no history of anxiety, depression, or other psychiatric or neurological disorders (Prins et al., 2006) no prior use of antipsychotic medication (Haider et al., 2023) demonstrated ability to read and understand research documents as assessed by researchers; and (Clayton, 2015) have no prior diagnosis of hearing impairment or demonstrate no abnormalities in preliminary hearing tests. The exclusion criteria were (Teodoro et al., 2018) schizophrenia diagnosis (Roy-Byrne et al., 2002) previously diagnosed emotional disorders with or without clinical treatment (Manu et al., 1988) drug addiction (Fukuda et al., 1994) severe organ dysfunction, and (Prins et al., 2006) diagnosed with hearing impairment or demonstrate abnormalities in preliminary hearing tests. Three hundred and thirty-five individuals were assessed for eligibility; 249 met the inclusion criteria and were enrolled (Figure 1).

### 2.2 Measures

The participants’ sociodemographic data were collected. Age, sex, and educational level were obtained at the first visit. All participants voluntarily sought treatment for the first time at the Neurology Department’s Fatigue and Depression Clinic. Participants were asked if they had had any infectious diseases such as colds, flu, and bacterial or viral infections before becoming ill, to which they answered “yes” or “no.” All eligible participants completed the following questionnaires.

#### 2.2.1 Symptom CheckList-90-Revised

Derogatis’ Symptom CheckList-90-Revised (SCL-90-R) is one of the most widely used measures of psychological distress (Derogatis, 1994). The inventory assesses a wide range of self-reported psychological problems and symptoms of psychopathology. SCL-90-R programs are simple, less invasive, and readily acceptable by patients early in the visit (Preti et al., 2019). Another advantage of SCL-90-R is its ability to distinguish and quantify psychosomatic symptoms from clinical manifestations (Achenbach et al., 2016). Each questionnaire item (90 in total) is scored on a five-point scale (0–4). It is then scored and interpreted based on nine primary symptom domains and three general indicators of psychological distress. The primary symptom dimensions include anxiety, depression, hostility, interpersonal sensitivity, obsessive-compulsive, paranoid ideation, phobic anxiety, psychotics, and somatization. The global indices comprise the global severity index, overall positive symptoms, and the positive symptom distress index. A total score exceeding 160 points suggests mild psychological distress, scores surpassing 200 points indicate moderate psychological issues, and scores exceeding 250 points demonstrate significant psychological difficulties.

#### 2.2.2 Hamilton Anxiety Rating Scale-14

The Hamilton Anxiety Rating Scale (HAMA) was presented as a rating scale for the severity of anxiety neurosis (Hamilton, 1959). The HAMA is a clinician-based questionnaire comprising 14 symptom-defined elements; it covers psychological and somatic symptoms, comprising anxious mood, tension, fears, insomnia, ‘intellectual’ (poor memory/difficulty concentrating), depressed mood (including anhedonia), somatic symptoms (including aches and pains, stiffness, bruxism), sensory (tinnitus, blurred vision), cardiovascular, respiratory symptoms (chest tightness, choking), gastrointestinal symptoms, genitourinary symptoms, autonomic symptoms (dry mouth, tension headache), and observed behavior during the interview (restless, fidgety). Each item is scored on an essential numeric scoring of 0 (not present) to 4 (severe); a score > 29 points is considered severe anxiety, >21 points indicates significant anxiety, >14 points indicates anxiety, 8–14 points indicates a tendency for anxiety, and ≤ 7 points indicates no anxiety. The general boundary value for the HAMA-l4 is 14 points.

#### 2.2.3 Hamilton Depression Rating Scale-24

The Hamilton Depression Rating Scale (HAMD), developed by Hamilton in 1960, is the most commonly used scale in the clinical evaluation of depression (Hamilton, 1960; Addington et al., 1996). The HAMD can be summarized into seven-factor structures: anxiety/somatization, weight, cognitive impairment, day and night change, blockage, sleep disorder, and sense of despair. We utilize the following severity ranges for the HAMD: no depression (0–7), a tendency for mild depression (Derogatis, 1994; Kennedy et al., 2005; Carruthers et al., 2011; Sáez-Francàs et al., 2012; Shungu et al., 2012; Cockshell and Mathias, 2013; Barnden et al., 2015; Xue and Liu, 2015; Achenbach et al., 2016; Klumpp and Shankman, 2018; Harrison et al., 2019; Preti et al., 2019; Wright et al., 2021), moderate depression (Hamilton, 1959, 1960; Addington et al., 1996; Wang et al., 2002, 2010; Cella et al., 2013; Frémont et al., 2013; Crawley, 2014; Faro et al., 2016; Jackson and Mac Leod, 2017; Salk et al., 2017; Herrera et al., 2018; Zhao, 2018; Qin et al., 2020), and severe depression (≥35).

### 2.3 SG P50 acquisition

Auditory evoked potentials were recorded using a 128-lead high-density electrophysiological EEG recorder MagstimEGI GES 300 provided by Magstim Inc. The recording electrodes were positioned according to the GSN-HydroCel-128, specifically targeting the Cz point. The impedances of all electrodes are below 50 kΩ. The experiment was conducted at a sampling frequency of 500 Hz. The experiment employed an auditory conditioned stimulus (S1) - test stimulus (S2) paradigm. The test took place in a shielded, soundproof room where participants sat in a relaxed, awake, and focused state. The background illumination was set to 2 lux. Prior to the test, participants received uniform instructions. A microcomputer program delivered groups of sound stimuli as conditioned stimulus S1 and test stimulus S2, with a 500 ms interval between stimuli and an intensity of 80 dB. Fifty groups were presented, separated by 10-s intervals. The input signal was amplified using an amplifier. The P50 component induced by the S1 stimulus was referred to as the conditioned stimulus wave (S1-P50), while the P50 component induced by the S2 stimulus was labeled as the test stimulus wave (S2-P50). Collected indicators included the amplitude (μV), and the amplitude difference and ratio between S1-P50 and S2-P50. Amplitude refers to the difference between the peak of the P50 wave and the preceding trough. The amplitude of both S1 and S2 is measured using this method. Previous studies have confirmed that the P50 SG ratio among healthy people typically falls below 50% (Wang et al., 2002). In this study, we chose a threshold of 50% to analyze the EEG changes in CFS patients based on this established norm.

MATLAB and the Net Station 4.3 version toolbox, an open-source software, were utilized for data pre-processing, which involved bandpass filtering from 0.1 to 30 Hz. Re-referencing was performed by averaging the values of all recording electrodes. Independent component analysis (ICA) was used to eliminate eye movements, and motion artifacts were manually removed. The epoch was extracted from 100 ms prior to the onset of S1 to 400 ms after the onset of S2. The initial 100 ms was utilized for baseline correction. The EEG data from each participant were averaged across all trials. The maximum positive peak, representing the P50 amplitude, was automatically extracted 30–90 ms after the onset of stimulation (Wang et al., 2010).

### 2.4 Statistical analysis

Statistical analyses were performed using SPSS V.26 (IBM, Armonk, NY, United States) and GraphPad Prism (version 9; GraphPad Software, San Diego, CA). Descriptive analyses were used to assess the demographic and clinical characteristics. General characteristics and P50 SG information were expressed as means and standard deviation. Spearman test was employed to assess correlations between variables. Furthermore, binary logistic regression analysis was performed for confirming the influence of various factors on P50 SG. All items on the scale were utilized, and various anxiety/depression groups were considered as factors of exposure to investigate their influence on the ratio of P50 SG. *p* < 0.05 were considered significant.

### 2.5 Grouping and abbreviations

#### 2.5.1 Grouping

In the subsequent subgroup analysis, anxiety and depression are assessed using HAMA and HAMD scales. Scores ranging from 0 to 7 on both scales indicate the absence of anxiety or depression. In HAMA, scores between 8 and 14 indicate the presence of anxiety, while in HAMD, scores between 8 and 20 indicate mild depression (Qin et al., 2020). Neither score can definitively indicate the presence of anxiety or depression; thus, we assign them similar interpretations within our subgroup analysis.

#### 2.5.2 Abbreviations

Only in HAMA: NA (0–7) - no anxiety; MHA (8–14) - may have anxiety; TMBA (15–20) - there must be anxiety; MBSA (21–28) - may be severe anxiety; TMBOA (≥29) - there must be obvious anxiety.

Combine HAMA and HAMD delimited by 7/7: NAOD (0–7 in both HAMD and HAMA) - no anxiety or depression; OA (0–7 in HAMD and ≥ 8 in HAMA) - only anxiety; OD (≥8 in HAMD and 0–7 in HAMA) - only depression; AAD (≥8 in both HAMD and HAMA) - anxiety and depression.

Combine HAMA and HAMD delimited by 14/20: NAOD (0–20 in HAMD and 0–14 in HAMA) - no anxiety or depression; OA (0–20 in HAMD and ≥ 15 in HAMA) - only anxiety; OD (≥21 in HAMD and 0–14 in HAMA) - only depression; AAD (≥21 in HAMD and ≥ 15 in HAMA) - anxiety and depression.

## 3 Results

### 3.1 General characteristics of participants

**Figure 2:**
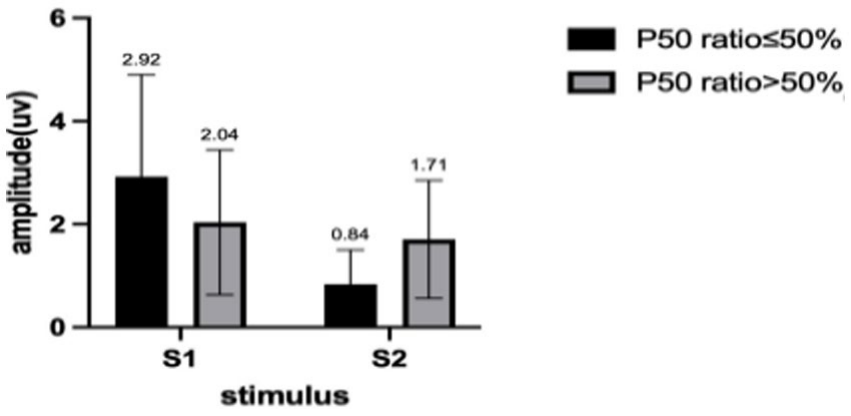
The mean and standard deviation of both sets of S1 and S2.

**Figure 3:**
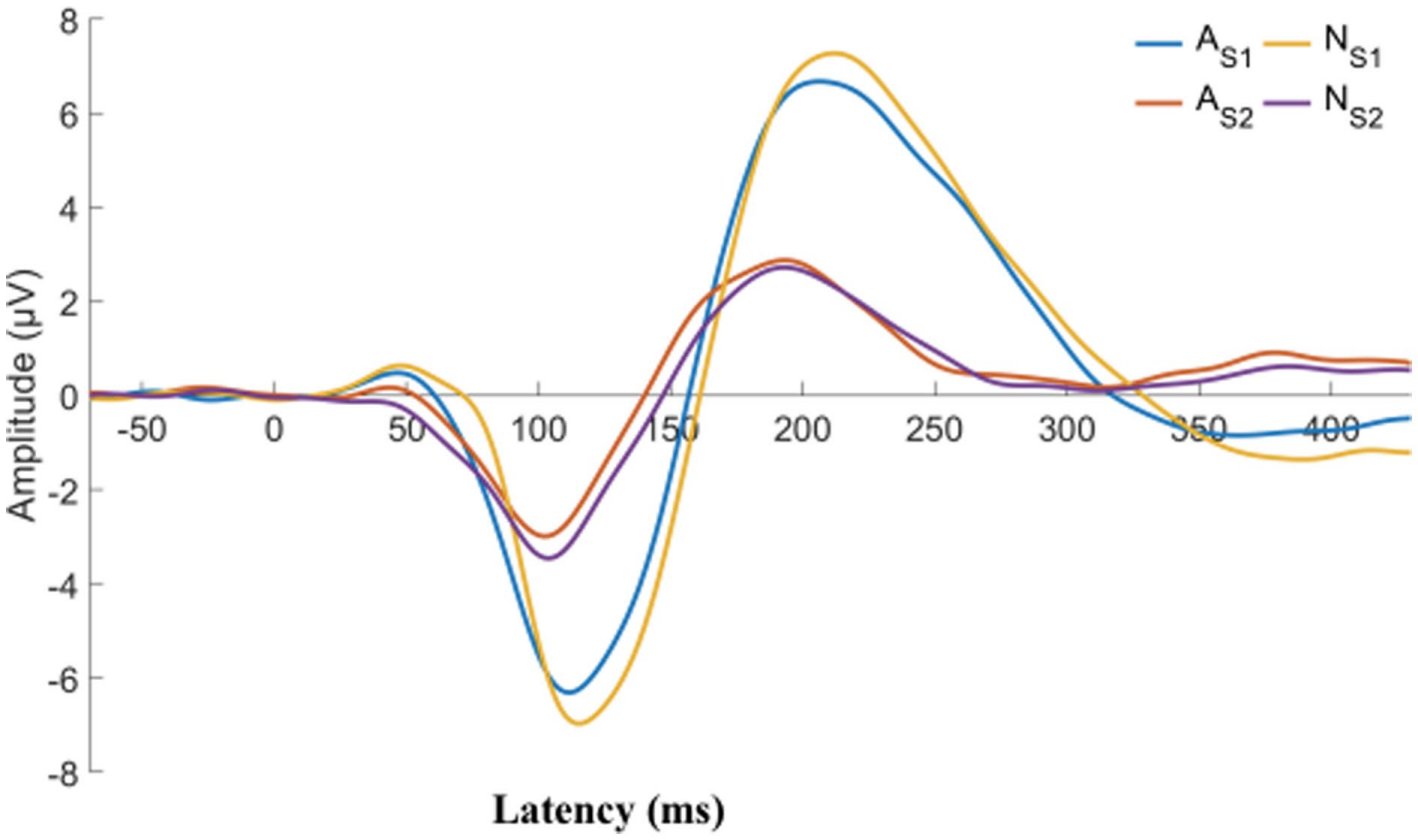
CFS patients were classified into two groups, normal and abnormal, based on their P50 SG ratio. The displayed graph illustrates the average S1 and S2 waveform for both groups. A, abnormal (P50 SG ratio > 50%); N, normal (P50 SG ratio ≤ 50%).

We describe the basic information and give a preliminary description of the degree of anxiety and depression and whether the P50 SG ratio is >50% (Table 1). The mean age of all subjects was 43.88 ± 15.47 years, and older patients (≥50 years old) accounted for the highest proportion (40.2%) during the age subgroups. A total of 43.8% of the subjects were male. Of the participants, 247 (99.1%) received education. Of these, 129 (51.8%) participants received undergraduate education or above. More than half of the subjects (56.6%) had an abnormal P50 SG ratio. All the participants were right-handed, and the EEG data was collected at the CZ (Central Zero) point; therefore, the results of P50 SG ratio in this study were not affected by this factor. The CFS patients were classified into two groups based on their P50 SG ratio: P50% ≤ 50and P50% > 50. We compiled the mean and standard deviation of S1 and S2 for both groups (Figure 2). The data from both groups was averaged and used to generate waveform and energy spectrum maps that highlighted significant differences between the two groups (Figures 3 4). The spectrum graphs shows that different P50 SG ratio characteristics of the two groups in certain brain regions (bilateral occipital, central parietal).

**Table 1:** Demographic and clinical characteristics of participants.

Individual characteristics	*n*	%
*Gender*		
Male	109	43.8
Female	140	56.2
*Age*		
≤30	58	23.3
31–40	54	21.7
41–50	37	14.9
≥50	100	40.2
*Educational level*		
Illiteracy	2	0.8
Primary school	8	3.2
Junior high school	32	12.8
Senior high school	36	14.5
Junior college	42	16.9
Undergraduate course	93	37.3
Postgraduate course	34	13.7
Doctor’s degree or above	2	0.8
*SCL-90*		
0–160	173	69.5
161–200	29	11.6
201–249	27	10.8
>250	20	8.0
*HAMD*		
0–7	52	20.9
8–20	83	33.3
21–34	88	35.3
>35	26	10.4
*HAMA*		
0–7	48	19.3
8–14	60	24.1
15–20	61	24.5
21–28	52	20.9
>29	28	11.2
*P50 SG ratio*		
≤50	108	43.4
>50	141	56.6

**Figure 4:**
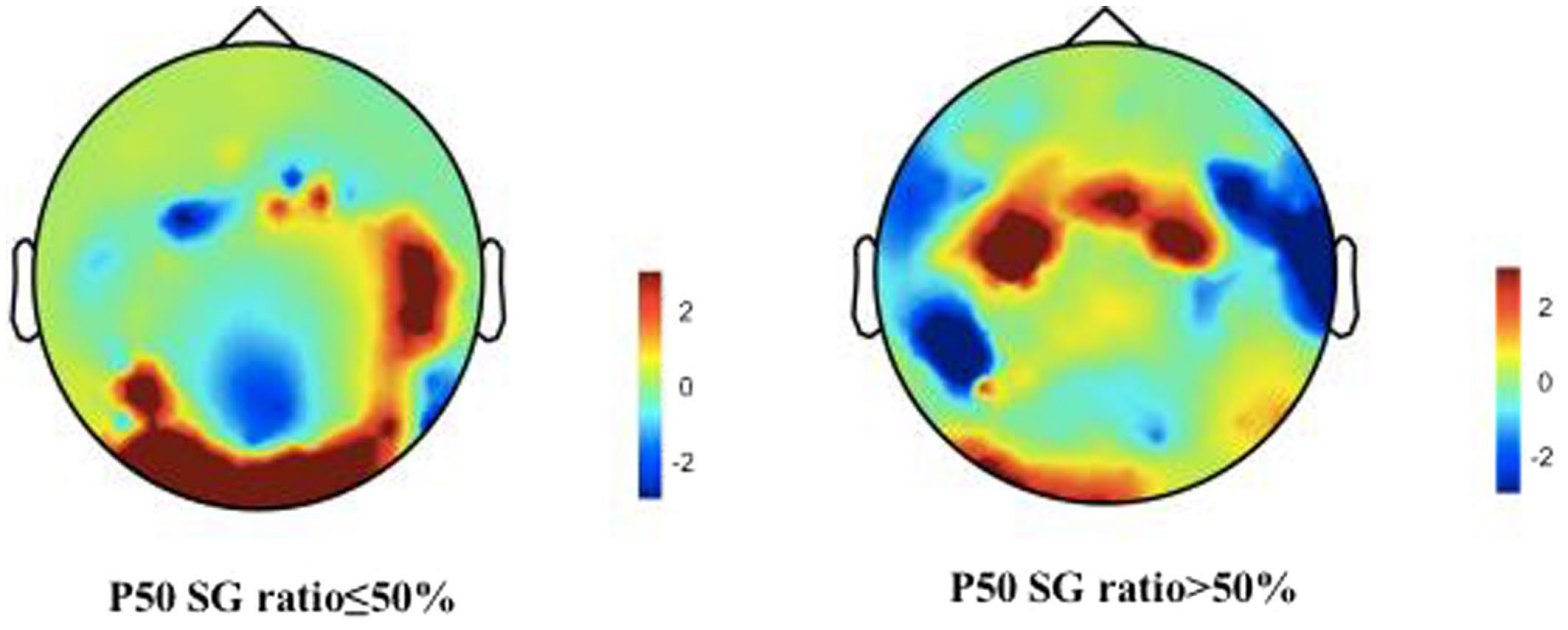
Brain activities in both normal and abnormal P50 SG ratio groups of CFS patients. A representation of “topographies” at the peak of P50 response illustrates the spatial distribution of brain activity, displays differences in mean energy spectral density. The diagram depicts the disparity in brain energy levels between the two groups. The figure showed that the energy accumulation in brain lobes may be different in both normal and abnormal P50 SG ratio groups of CFS patients.

**Figure 5:**
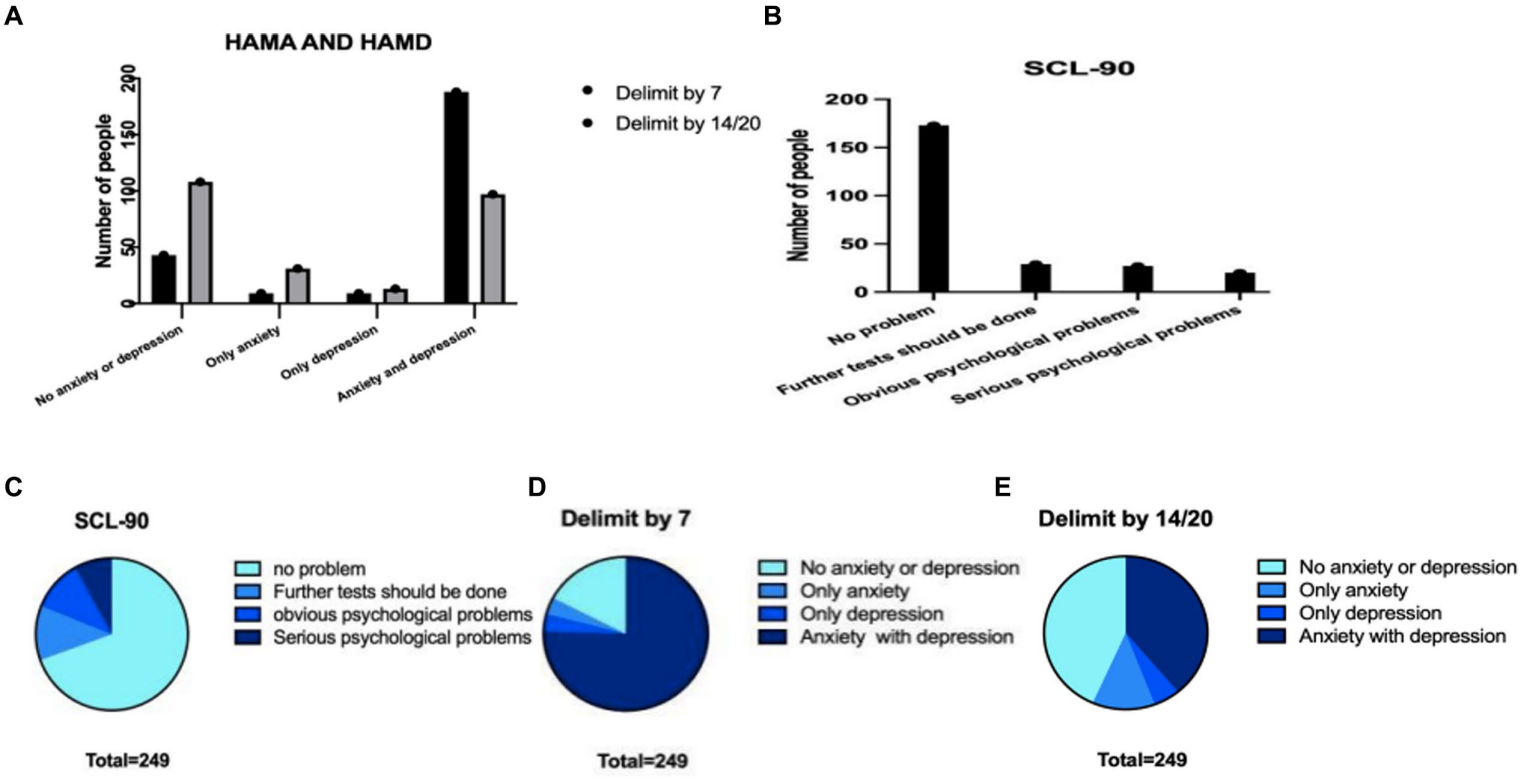
The characteristics of the three scales in the total population were compared with the data differences obtained from different scales. **(A)** Number of HAMA and HAMD patients with different dividing lines **(B)** number of patients with different SCL-90 scores **(C)** proportions of different parts of SCL-90 **(D)** the proportion of patients with different degrees of HAMA and HAMD scales divided by 7 **(E)** the proportion of patients with different degrees of HAMA and HAMD scales divided by 14.

Since in this version of HAMA and HAMD, 0–7 points both represent absolute absence of anxiety/depression, 8–14 points may have anxiety in HAMA, 8–20 may have depression in HAMD, we choose 7/7 for the first and 14/20 for the second analysis. With the score of 7/7 as the threshold for anxiety/depression, there were 43 patients without anxiety or depression, accounting for 17.3%. If we saw reference to score of 14/20, there were 108 patients without anxiety/depression, accounting for 43.4%. However, based on the results of SCL-90-R, among those 173 people here, 69.5% showed no mental problems (Figure 5). Then, we did a grouping analysis. Before this step, we conducted normality analysis on all continuous variables, and all continuous variables did not satisfy normal distribution.

We also conducted non-parametric tests on the P50 SG ratio of different variable groups (Table 2). Normal distribution was not met after grouping owing to the small sample size of some groups and subgroups. We chose the Kruskal–Wallis test of the non-parametric tests for comparisons of significant differences in the P50 SG ratio between different groups. The results showed no significant difference in P50 SG ratio between age, sex, HAMD, SCL-90, and anxiety/depression groups divided by 7/7 (*p* > 0.05, Table 2).

**Table 2:** Non-parametric tests for differences in P50 values between different variable groups.

The K-W test	Total = 249	*N* (%)	Degrees of freedom	*p* value
Age	The youth	58 (23.3)	3	0.764
	Young and middle-aged	54 (21.7)		
	The middle-aged	37 (14.9)		
	The middle-aged and elderly	100 (40.2)		
Gender	Male	109 (43.8)	1	0.079
	Female	140 (56.2)		
Anxiety (HAMA)	No anxiety	48 (19.3)	4	*0.032*
	May have anxiety	60 (24.1)		
	There must be anxiety	61 (24.5)		
	There must be obvious anxiety	52 (20.9)		
	May be severe anxiety	28 (11.2)		
Depression (HAMD)	No depression	52 (20.9)	3	0.055
	Maybe mild depression	83 (33.3)		
	Must be depressed	88 (35.3)		
	Severe depression	26 (10.4)		
SCL-90	No problem	173 (69.5)	3	0.353
	Further tests should be done	29 (11.6)		
	Obvious psychological problems	27 (10.8)		
	Serious psychological problems	20 (8.0)		
Anxiety/depression(divided by 7)	No anxiety and depression	43 (17.3)	3	0.092
	Only anxiety	9 (3.6)		
	Only depression	9 (3.6)		
	Anxiety with depression	188 (75.5)		
Anxiety/depression (divided by 14/20)	No anxiety and depression	108 (43.4)	3	*0.024*
	Only anxiety	31 (12.4)		
	Only depression	13 (5.2)		
	Anxiety with depression	97 (39.0)		

*p* < 0.05 in italics was considered to be different between groups.

However, in the HAMA and anxiety/depression groups divided by 14/20, some groups had significant differences in the P50 SG ratio (*p* < 0.05, Table 2). The P50 SG ratio of the HAMA group were significantly different between the NA and TMBOA groups (*p* < 0.05, Table 3). There was a significant difference in the P50 SG ratio between the NAOD and AAD groups with the threshold of 14/20 (*p* < 0.05, Table 4).

**Table 3:** Pairwise comparison for the P50 SG ratio of anxiety degree groups.

Sample 1-sample 2	Test statistics	Standard error	Standard test statistics	Significance	Adj. significance^a^
NA-MHA	−16.796	13.946	−1.204	0.228	1.000
NA-TMBA	−16.973	13.895	−1.222	0.222	1.000
NA-MBSA	−32.926	17.126	−1.923	0.055	0.545
NA-TMBOA	−44.037	14.415	−3.055	0.002	*0.023*
MHA-TMBA	−0.177	13.094	−0.014	0.989	1.000
MHA-MBSA	−16.130	16.483	−0.979	0.328	1.000
MHA-TMBOA	−27.241	13.645	−1.996	0.046	0.459
TMBA-MBSA	−15.953	16.439	−0.970	0.332	1.000
TMBA-TMBOA	−27.064	13.593	−1.991	0.046	0.465
MBSA-TMBOA	11.111	16.881	0.658	0.510	1.000

Each row tests the null hypothesis that sample 1 and sample 2 have the same distribution. Progressive significance was shown (two-sided test). The significance level was 0.05. ^a^Significance values have been adjusted by Bonferroni correction for a number of tests. NA, no anxiety; MHA, may have anxiety; TMBA, there must be anxiety; MBSA, may be severe anxiety; TMBOA, there must be obvious anxiety. *p* < 0.05 in italics was considered to be different between groups.

**Table 4:** Pairwise comparison for the P50 SG ratio of anxiety and depression groups (divided by 14/20).

Sample 1-sample 2	Test statistics	Standard error	Standard test statistics	Significance	Adj. significance^a^
NAOD-OD	−16.220	14.674	−1.105	0.269	1.000
NAOD-AAD	−29.949	10.074	−2.973	0.003	*0.018*
NAOD-OA	−30.307	21.142	−1.433	0.152	0.910
OD-AND	−13.729	14.859	−0.924	0.356	1.000
OD-OA	−14.087	23.796	0.423	0.554	1.000
AAD-OA	0.358	21.270	0.335	0.987	1.000

Each row tests the null hypothesis that sample 1 and sample 2 have the same distribution. Progressive significance was shown (two-sided test). The significance level was 0.05. ^a^Significance values have been adjusted by Bonferroni correction for a number of tests. NAOD, no anxiety or depression; OA, only anxiety; OD, only depression; AAD, anxiety and depression. *p* < 0.05 in italics was considered to be different between groups.

Finally, we conducted a correlation analysis, which analyzed the correlation between the P50 SG ratio and the variables of each scale (Table 5). We also conducted multi-angle analysis by grouping (Table 5). We discussed the correlation between the P50 SG ratio and other variables from the total sample, the normal P50 SG ratio sample, the abnormal P50 SG ratio sample, and the anxiety/depression sample divided by 7/7 and 14/20. In the overall sample, the somatization scores in the HAMA and HAMD were correlated with the P50 SG ratio, and the somatization scores in HAMD were associated with P50 SG ratio in the OD group delimited by 14/20 and the AAD group delimited by 7/7 and 14/20 (*p* < 0.05, Table 5). In the NAOD group delimited by 14/20, the somatization scores in HAMA are also associated with the P50 SG ratio. Among the general population, sense of despair, hostility, and paranoid ideation were correlated with the P50 SG ratio (*p* < 0.05, Table 5). Regarding the correlation between P50 SG ratio and anxiety and depression symptoms that this study focuses on, the overall characteristics of the results of other groups are as follows: the greater the levels of anxiety and depression symptoms, the greater the correlation is. When comparing the 14/20 groups with the 7/7 groups, the correlation is greater with the group exhibiting more symptoms (Table 5).

**Table 5:** The correlation analysis results of P50 value and various variables.

Spearman nonparametric test			Total (*N* = 249)	P50 normal (*N* = 108)	P50 abnormal (*N* = 141)	AAD delimited by 7 (*N* = 188)	NAOD on delimited by 14/20 (*N* = 108)	OD delimited by 14/20 (*N* = 13)	AAD delimited by 14/20 (*N* = 97)
	Somatic symptoms	Sig. (Double tail)	0.012^*^				0.022^*^		
	Psychological symptoms	Sig. (Double tail)	0.003^**^			0.035^*^	0.023^*^		0.030^*^
	HAMA total points	Sig. (Double tail)	0.002^**^			0.025^*^	0.013^*^		0.040^*^
	Anxiety/somatization	Sig. (Double tail)	0.002^**^			0.004^**^		0.048^*^	0.016^*^
	Weight	Sig. (Double tail)		0.033^*^					
	Cognitive impairment	Sig. (Double tail)				0.003^**^	0.009^**^		0.006^**^
	Day and night change	Sig. (Double tail)			0.032^*^				0.038^*^
	Blockage	Sig. (Double tail)			0.039^*^	0.002^**^	0.017^*^		0.003^**^
	Sense of Despair	Sig. (Double tail)	0.001^**^			0.002^**^	0.044^*^		0.013^*^
	HAMD total points	Sig. (Double tail)	0.001^**^			0.001^**^			0.003^**^
	Interpersonal sensitivity	Sig. (Double tail)						0.038^*^	
	Depression	Sig. (Double tail)						0.036^*^	
	Hostility	Sig. (Double tail)	0.018^*^					0.048^*^	
	Paranoid ideation	Sig. (Double tail)	0.019^*^						

^*^At level 0.05 (two-tailed), the correlation was significant. ^**^At level 0.01 (two-tailed), the correlation was significant. NAOD, no anxiety or depression; OA, only anxiety; OD, only depression; AAD, anxiety and depression.

In the regression analysis of each item on the three scales, solely the hopelessness score in HAMD emerged as a risk factor for an abnormal P50 SG ratio. However, other factors showed no significance. The following binary logistic regression analysis of P50 SG ratio showed that the OA and AAD groups delimited by 14/20 in different groups significantly influenced the P50 SG ratio (*p* < 0.05, Table 6).

**Table 6:** Binary logistic regression analysis.

Variable in the equation									
		*B*	Standard error	Wald	Degree of freedom	Significance	Exp(B)	95% confidence interval for EXP(B)	
								The upper limit	The floor limit
Step 1a	NAOD			8.155	3	0.043			
	OA	−0.802	0.287	7.793	1	*0.005*	0.448	0.255	0.787
	OD	−0.552	0.418	1.748	1	0.186	0.576	0.254	1.305
	AAD	−0.147	0.609	0.058	1	0.809	0.863	0.262	2.846
	Constant	0.617	0.213	8.400	1	*0.004*	1.853		

NAOD, no anxiety or depression; OA, only anxiety; OD, only depression; AAD, anxiety and depression. ^a^Variables entered in step 1: anxiety and depression group. *p* < 0.05 in italics was considered to be different between groups.

## 4 Discussion

The concept of CFS continues to receive widespread attention, and fatigue is becoming a major public health problem. Its etiology and pathogenesis remain unclear, resulting in a lack of specific treatments, possibly due to the involvement of multiple systems and difficulty distinguishing CFS symptoms from those of anxiety and depression (Zhao, 2018). To our knowledge, few studies have investigated the relationship between fatigue and anxiety/depression. This cross-sectional study analyzes anxiety/depression in a population of patients with CFS. It explores the distribution characteristics of anxiety/depression symptoms in this population and the correlation of the P50 SG ratio with different symptoms.

The incidence rate of CFS varies significantly according to country, sex, age, and educational background (Crawley, 2014; Faro et al., 2016; Herrera et al., 2018). Our results show that CFS is more common in women (56.2%), people older than 50 years old (40.2%), and people with relatively high education levels, similar to the characteristics of previous findings (Frémont et al., 2013). The participants were highly educated and more concerned about their health, which may explain why they actively sought help in the fatigue clinic of the general hospital.

The fatigue dimension of CFS patients exists in the physical and mental aspects, mainly manifested as anxiety, depression, irritability, and emotional instability (Cella et al., 2013; Jackson and Mac Leod, 2017; Salk et al., 2017). We first evaluated the SCL-90 scale for this population. SCL-90 has a comprehensive dimension and a specific ability to distinguish physical and mental symptoms, which has good reliability and validity in Chinese populations (Chen and Li, 2003; Sun et al., 2017; Dang et al., 2021). The results of the SCL-90 scale among the outpatient population indicated a comparatively low prevalence of psychological issues and a comparatively high prevalence of physical or somatic symptoms. These findings prompt our hypothesis that CFS may manifest as a distinct condition, separate from anxiety, depression, and other emotional disorders. Some patients with CFS may solely experience physical symptoms without comorbid emotional symptoms. After checking the widely used the HAMA and HAMD (Zhang et al., 2020; Zimmerman et al., 2020), we observed that the data from the HAMA, HAMD, and SCL-90 exhibited distinct trends regarding the assessment of emotional disorders (Figure 5). This confirmed that most patients with CFS had anxiety (69.5%) and depression (79%) according to HAMA and HAMD, suggesting that people with CFS have a high rate of comorbidity with anxiety/depression (Leong et al., 2022). While many patients experience noticeable mental symptoms like anxiety or depression, they tend to perceive their condition primarily in terms of physical problems. This perception aligns with the clinical profile of most of these patients, who initially seek treatment at non-psychiatric hospitals. The SCL-90 results reflect that most patients present for physical symptoms such as fatigue without revealing obvious psychiatric problems, which is not inconsistent.

We now discuss the possible reasons for this different trend between those scales. The HAMA and HAMD are rating scales of the physician’s assessment, while the SCL-90 is a self-rating scale. There are differences in the evaluation subjects. In addition, the scales have specific differences in the classification of different symptoms, which may be one of the reasons for the difference in results (Carrozzino et al., 2020). Moreover, the characteristics of our outpatients are, the general hospital neurology outpatients some clinicians consider psychological problems, patients do not recognize, they usually feel that they are the existence of physical disease. This phenomenon has been reported in the literature (Wang et al., 2022).

Although the exact underlying mechanism of fatigue in individuals with CFS remains unclear, it is believed to involve the central fatigue predominantly, as this is primarily associated with stress (Aleksandrov et al., 2016). However, the pathogenesis of CFS in cases associated with other factors, such as viral infection, may be more intricate. Some studies have demonstrated that muscle fatigue affecting the central fatigue will further weaken the SG (Evstigneeva et al., 2010; Aleksandrov et al., 2016). The normal brain filters out non-important information and selects useful information. Through this gating, valuable information is absorbed and transmitted to the higher brain to avoid an overload of information intake (Wang et al., 2009). The S2 to S1 ratio is the commonly used metric to separate patients from controls (Smith et al., 1994; Jiang et al., 2015; Xie and Liang, 2015). In the present study, more than half of the population (56.6%) had an abnormal P50 SG ratio. In our total sample, correlation analysis revealed that the P50 SG ratio was associated with somatization symptoms in HAMA and HAMD, suggesting a potential correlation between P50 SG and fatigue (*p* < 0.05, Table 5). Patients with CFS with no anxiety/depression delimited by 7/7 showed no apparent correlation between P50 SG and somatic symptoms. However, when the grouping method was modified by adjusting the cutoff to 14/20 and adding patients with potential symptoms of anxiety/depression, a correlation was observed. These findings suggest that as the symptoms of anxiety/depression worsen in CFS patients, the degree of brain function abnormalities becomes increasingly apparent. This was initially attributed to an association with anxiety/depression, but it is more reasonable to associate it with physical symptoms. Since there is no reliable measure for evaluating fatigue, the data in this study cannot provide further insights into the relationship between fatigue and physical symptoms. However, in scale classification, fatigue is categorized as a physical symptom. Therefore, the presence of P50 SG ratio abnormalities in these patients should be interpreted as an indication of increasingly pronounced brain function abnormalities as anxiety/depression and fatigue worsen in patients with CFS.

Previous studies have reported that abnormalities in P50 SG ratio are likely associated with anxiety and depression in patients (Xie and Liang, 2015). Our data further support these findings. The P50 SG ratio in patients with schizophrenia is influenced by depression (Li et al., 2023). The literature has shown that P50 SG ratio levels are correlated with anxiety, depression, and cognitive function (Sussman et al., 2014). In our study, regression analysis showed that among the influencing factors in different anxiety/depression groups, the OA and AAD groups delimited by 14/20 impacted P50 SG ratio. The abnormal influence of P50 SG ratio may contribute to dysregulated brain function, potentially leading to heightened anxiety/depression in certain patients. This study revealed additional dimensions of the HAMA and HAMD, including cognition, fear, hostility, and paranoia (Table 5). With the subgroup of NAOD going from 7/7 with no anxiety and depression symptoms at all to 14/20 with possible anxiety and depression symptoms, the percentage of patients with abnormal P50 SG ratio responses increased from 17.3 to 43.4%, the symptoms of anxiety and depression were significantly intensified, and a significant correlation was observed. It is plausible that with the progression of the disease, abnormal brain function manifests an increasing number of psychiatric symptoms. Furthermore, among the 43 patients in the NAOD group delimited by 7/7, 20 tested positive for P50 SG ratio (data not provided in Table 2), the differences in P50 SG ratio degree observed may not solely be attributable to anxiety and depression, but may also be significantly associated with the sleep problems among patients suffering from CFS, which this study was not addressed. These previously unexplored characteristics of the NAOD groups offer new avenues for investigation. Our findings demonstrate the possible application of the objective P50 SG test to differenate CFS patients with anxiety or depression (Figure 4).

SG of P50 auditory-evoked potentials has been previously suggested in other literature to contribute to stimulus selection and information processing and may further affect cognition and emotion. The specific mechanism may be related to cholinergic regulation and hippocampal-mediated preattention channels (Adler et al., 1993; Freedman et al., 1994, 1996). P50 SG inhibition may involve activity across brain networks. Previous experiments have found that P50 SG ratio can change the rhythm of the entire cerebral cortex with the reduction of beta waves (Javitt and Freedman, 2015), and a higher P50 SG ratio than normal indicates a weaker ability to process information. This could be the underlying pathophysiological mechanism responsible for the abnormal P50 SG ratio observed in patients with CFS.

This study had some limitations. First CSF patients with anxiety/depression history (diagnosed and treated before they were recruited in our study) were not enrolled. All of them were treated with certain kinds of medications for their symptoms, which could disturb the measurement of P50 SG (Oranje et al., 2011). In addition, we only got few patients of this kind (total number was less than 5). However, excluding these patients may affect the evaluation of correlation coefficient.

The jumpy nature of the scale scoring method hampers accurate grading of symptom severity, leading to a decrease in grouping accuracy. Moreover, the data exhibited poor normality, thereby impacting the analysis of the results to an extent. Furthermore, This study employs a cross-sectional design, focusing on symptoms and EEG physiological markers among individuals diagnosed with CFS. Further exploration of their precise relationships necessitates additional cohort studies. The absence of reliable fatigue symptom scales or other measurement tools also hinders further research on CFS.

Considering the intricate etiology of CFS and the nature of symptom-based diagnostic criteria, some heterogeneity among the patients included in this study was expected. Nevertheless, most patients exhibited discernible abnormalities in P50 SG ratio that were closely associated with clinical symptoms, encompassing fatigue, emotion, cognition, and overall well-being.

The findings of this study indicate that abnormal brain function potentially plays a vital role in CFS pathogenesis. Furthermore, a positive correlation between the number of symptoms exhibited by the P50 index and clinical symptoms was observed, reinforcing the significance of this association. Consequently, in light of the study findings, we posit that CFS patients exhibiting the higher P50 SG ratio should be categorized into distinct subtypes or subgrouped within the functional neurological disorders spectrum to promote additional exploration of their central fatigue mechanisms.

## Data availability statement

The original contributions presented in the study are included in the article/supplementary material, further inquiries can be directed to the corresponding author.

## Ethics statement

The studies involving humans were approved by The Human Research Ethics Committee of Beijing Friendship Hospital, affiliated with Capital Medical University. The studies were conducted in accordance with the local legislation and institutional requirements. Written informed consent for participation was not required from the participants or the participants’ legal guardians/next of kin in accordance with the national legislation and institutional requirements.

## Author contributions

XL: Writing – original draft, Data curation, Formal analysis, Investigation, Methodology, Supervision, Validation, Writing – review & editing. SL: Data curation, Formal analysis, Visualization, Writing – review & editing. RR: Data curation, Writing – review & editing. XW: Formal analysis, Writing – original draft, Writing – review & editing. CH: Writing – review & editing. ZL: Writing – review & editing.
